# Supplementary material for: Chromothripsis during telomere crisis is independent of NHEJ, and consistent with a replicative origin
Source: Genome Res. 2019 May;29(5):737–49. doi: 10.1101/gr.240705.118 (PMC6499312; doi:10.1101/gr.240705.118)
Supplement: Supplemental Material [file supp_gr.240705.118_Supplemental_file_1.zip › contigs/annotated_contigs/DB53/contig.3.DB53_length_1022_mean_cov_10.1428571429.docx]

**DB53_length_1022_mean_cov_10.1428571429**

```
TAATATAGAAATAAAGTGCACAATAAATGTAATGTGCTTGAATCATCTGAAGCCATCCCCCACCCTGCCCTCTTGGTACATGGAAAAAT
 >chr8:136796207-136796682 + E=1e-270
TGTCTTCCATGAAACCAGTCCCTGGTGCCAAAAAGGTTGGGGAAATGCTGACTTACACAACAGAAATTTATTTGCTAAGTTTTGAAGGC

TGGAAGTTCAGGATCAAGGTTCTGGCAAACTTATTAGCTTTTCCTCCTTCCCTGTCTCACCATTCCTTTCTTCTGCTTCCTGTGATCAC

CTTTTGAACTGTTTTTGCAAATTTATGTCTTGGGCTTTGCTCTTAAGGAAATCTAAAATGAGACACCATAGTTTTAGAAAAAGCCGAAT

AGTTTCTCTTTTTGAGAGTTCTCATAGAGTTTTATCAACTATTTTGGATTATACTTATCTAATTACGGTATGTTTTGGCCACTGAACCT

TTTAAAATGTATTATCTTGAAAGCATGT|GC|ATTCTAGGTGCTCTATGTCCTCGTCAGCACTTGATATTGTCAGATTTTGTGTTGTGT
                             >chr17:45037582-45037753 - E=7e-91
TTGTTTGTATGTATGCCAAGTGTGTAGTGACACCTCATTGTGTGTCTAATTTGTCTTCTATTGAATGATGATGTTGAGCATCTTTTCAT

GTTCTTAAGTGCCATCTGTATAT|ATGTGCTCACCGGGGAGCCACGGGTTGGGTATCTTCCTTCTCCATCCTGAGAGTCAGAGGCTGTC
                         >chr7:154886918-154887296 + E=2e-215
ATCTTGCCTCGTGGTGGTGGCACAACCTCTCCCCCAACCTGCAGGTCCCTGTTCCTCCACTGTCGTCTTAGCCACTCCTGTGCCAGTTT

TGTCTTGTACTTTACTGCCTGTTGGGCAAGGCAATGTGCCATGGTCCCTGAGCCACCTGCACATGCTTGCTGACCATGCCAGGAATGCA

AGACCCTTCTTTCTCTTTGACAAAGCATGAGCACCGCCATCTTGGACAAACACCACTGTTCTAAGTTCCCCTTGATTTAAAAACTGCCT

AAATCCAGCCCCCAAACATCAGCCTAATGGCTAATGTCAACATAAC
```
